# Time Series Analysis of Muscle Deformation During Physiotherapy Using Optical Wearable Sensors

**DOI:** 10.3390/s25113507

**Published:** 2025-06-02

**Authors:** Satoshi Shimabukuro, Tamon Miyake, Emi Tamaki

**Affiliations:** 1 Faculty of Engineering, University of the Ryukyus, Okinawa 903-0213, Japan; 2 Department of Physical Therapy, Okinawa Rehabilitation Welfare College, Okinawa 901-1301, Japan; 3 H2L Inc., Tokyo 106-0032, Japan; 4 Future Robotics Organization, Waseda University, Tokyo 169-8050, Japan; 5 Department of Systems Innovation, School of Engineering, The University of Tokyo, Tokyo 113-8656, Japan

**Keywords:** time series analysis, optical sensor, muscle deformation, physiotherapy, novice and expert

## Abstract

Wearable devices are used to acquire and analyze biometric information. However, the lack of consensus on standardized devices and analytical methods for representing the time series data of muscle force and coordination has hindered the interpretation and comparison of such data across studies. This study aimed to compare time series data between novices and experts during physiotherapy sessions to identify differences in the degree of force and coordination. Optical wearable muscle deformation sensor arrays were used to capture muscle bulging (muscle deformation) and visualize the force levels. Two types of physiotherapy sessions were conducted in the upper and lower limbs, and the time series data of muscle deformations collected during these sessions were analyzed using visualization, autocorrelation coefficients, and cross-correlation analysis. Although differences were observed visually and in effect sizes, no statistically significant group differences remained after covariate adjustment. The results revealed differences in the magnitude of force and muscle coordination. These findings highlight measurable distinctions in muscle activation patterns and coordination strategies between novices and experts, suggesting potential applications for training and skill evaluation in physiotherapy and related domains.

## 1. Introduction

### 1.1. Background

In recent years, wearable technology has revolutionized many fields by enabling the objective and continuous monitoring of physical motor functions [1]. In sports [2] and fitness [3], wearable devices help to optimize performance and improve exercise efficiency [4,5]. Wrist-worn wearables have become widely used, providing easy access to physical data such as heart rate and step count [6]. In medicine and rehabilitation, wearable technology provides critical data on posture, motor control, biomechanics, and the evaluation of treatment effects, aiding patient recovery [7]. In addition, wearable devices are increasingly being used in the education of rehabilitation professionals to enhance skill acquisition [8]. Many studies have reported the use of accelerometers [9] and electromyography devices [10] in rehabilitation training. However, applying an appropriate degree of force during patient interactions is crucial for adjusting the treatment difficulty and effectiveness. Marked differences in force control between experts and novices have been reported [11]. Measuring and analyzing time series data are essential for accurately assessing these differences [12]. Time series data analysis is not limited to static assessment, but can capture dynamic changes in muscle strength and movement patterns over time. This is important for improving patient outcomes, skill acquisition, and performance assessments during rehabilitation. In rehabilitation, approaches combining surface EMG and multichannel EMG technology have been widely applied to evaluate objective muscle activity [13] indicators for biofeedback training [14] and educational support. For example, the introduction of EMG education for physiotherapy students has attracted attention in the field of education and skill transfer, and there are examples of improved student understanding of exercise by incorporating sEMG practice into the curriculum [15]. The use of EMG sensors in rehabilitation is increasing; however, they suffer from motion artifacts and require complex preprocessing [16]. In contrast, muscle deformation detection has the potential to represent mechanical muscle activity directly and with less noise. Recent advancements in optical sensing, such as light myography, have demonstrated improved robustness and decoding accuracy compared to traditional sEMG, particularly in gesture recognition tasks [17]. For example, Shahmohammadi et al. demonstrated that light myography outperformed sEMG in gesture decoding accuracy, while Franco et al. reported strong temporal correlations (r > 0.85) between optical finger signals and clinical EMG during upper limb rehabilitation tasks [18]. These findings support the feasibility of optical approaches to capture physiologically relevant muscle signals. Building upon this foundation, wearable muscle deformation sensors, which detect surface displacements associated with underlying muscle contractions, offer a promising extension of optical methods by directly measuring the mechanical changes of muscle bulging. However, it remains unclear how such muscle deformation signals vary over time in clinical settings, such as physiotherapy, although there have been reports of differences in muscle deformation with skill level [19]. Few studies have quantitatively captured these differences in time series muscle behavior using wearable technology. In physiotherapy, observational checklists and subjective performance ratings are commonly used to distinguish novice and expert movements [20]. However, these methods are inherently limited by rater bias, inter-rater variability [21], and a lack of temporal resolution. Traditional motion analysis tools, such as goniometry and video-based tracking, provide valid kinematic data, but often fail to detect subtle differences in neuromuscular control that distinguish skill levels [22]. Furthermore, although sEMG is widely employed, it is susceptible to signal cross-talk and skin impedance, which limits its reliability in fine motor assessments [23]. Therefore, there is a growing need for objective, high-resolution, sensor-based methods capable of continuously capturing physiological muscle behaviors during exercise tasks. A sensor-based assessment framework that enables a continuous and non-invasive characterization of skill-related muscle control patterns could address this gap.

### 1.2. Related Works

Electromyography (EMG) and optical myography sensors (optical sensors) are widely used to assess muscle activity as biosignals in physiological and clinical research. EMG measures the electrical potentials generated by muscle fibers during contraction, providing direct insight into neuromuscular activation patterns [24,25,26]. However, EMG’s reliance on surface electrodes makes it susceptible to variations in muscle length (sarcomere length) and electrode–skin impedance, which fluctuate with joint angle and perspiration. These factors necessitate precise anatomical placement and extensive preprocessing (rectification, smoothing) to estimate force, and even then, EMG struggles to isolate deep muscle activity due to signal cross-talk [27]. Moreover, EMG readily picks up electromagnetic interference from fluorescent lighting, power cables, and electronic devices, compromising signal quality in real-world or ambulatory settings. In contrast, OMG uses near-infrared light to detect mechanical deformation of muscles via changes in skin reflectance [17]. When paired with an inertial measurement unit (IMU), an optical sensor array can directly capture muscle bulging while compensating for joint angle-induced changes in muscle geometry, greatly enhancing robustness to posture variations. Because OMG relies on diffuse light reflection rather than biopotentials, it remains relatively stable even under wet conditions; perspiration, which often short-circuits EMG electrodes, minimally impacts optical measurements. Likewise, infrared-based optical sensors are essentially immune to the electromagnetic noise that plagues EMG. Finally, optical sensors require no skin preparation or precise electrode placement: a simple band can be donned in seconds, enabling rapid deployment in clinical or sports environments without the logistical burden of EMG setups. Despite some sensitivity to ambient lighting and skin properties (e.g., pigmentation, moisture), these limitations can be mitigated through controlled illumination and calibration, making optical myography a convenient tool for continuous, wearable muscle monitoring [28,29].

### 1.3. Objective

**Figure 1:**
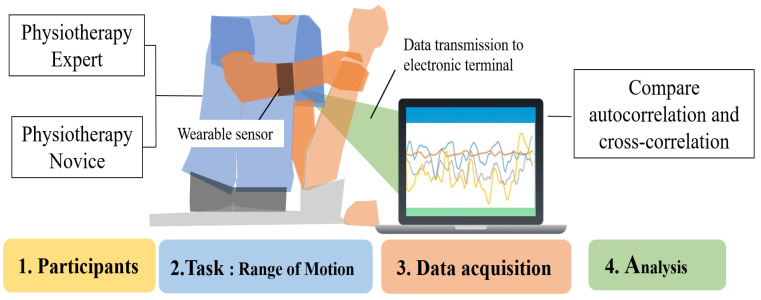
Task analysis of experts and novices using an optical wearable device.

This study used wearable sensors to acquire time series data on forearm muscle deformation during physiotherapy, and differences between novices and experts were analyzed. By comparing force variations over time, the analysis aims to identify technical characteristics unique to experts and contribute to the enhancement of learning effectiveness for novices (Figure 1). When comparing novices and experts, experts are generally considered more stable and efficient in their treatment.

Two key differences between novices and experts are hypothesized: (1) Experts exhibit more stable muscle control during physiotherapy than novices. (2) Experts show an inverse relationship in muscle deformation between active and antagonistic muscles, compared to novices.

## 2. Materials and Methods

### 2.1. Participants

The participants included ten first-year students enrolled in a physiotherapy training school and ten physiotherapists with more than ten years of experience (Table 1). The students were required to have no practical experience related to physiotherapy and less than one month of work experience in a medical setting. Physiotherapists were assumed to have a history of teaching in a physiotherapy school and a degree or certification as a physiotherapy specialist.

The study participants had to meet the following criteria: they had completed medical treatment and rehabilitation, and were not regularly taking any medications prescribed by a physician; had no severe pre-existing medical conditions; had no cold-like symptoms, injuries, or muscular discomfort that would impair their ability to exercise on the day of the experiment. In addition, they must have slept at least 7 h the day before and the day of the experiment and must not have consumed alcohol. Any condition that was inappropriate for the experiment or required special consideration led to exclusion from participation.

### 2.2. Equipment

**Figure 2:**
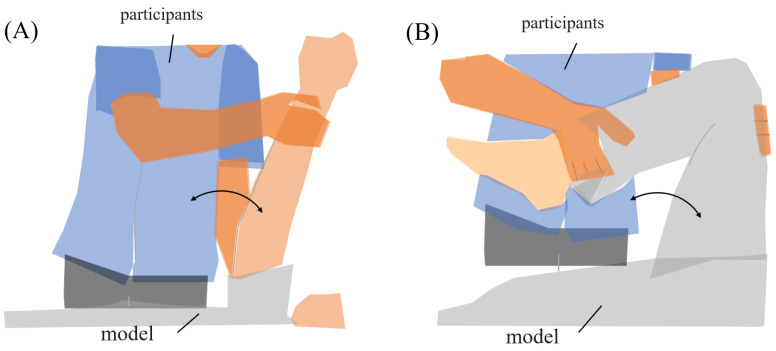
Range of motion for experimental tasks. (**A**) Upper limb raising exercise. The subject moved the model’s arm from elbow extension to 90° shoulder flexion, then returned it to the original position. (**B**) Lower limb flexion exercise. The subject moved the model’s lower limb from extension to 90° knee flexion, then returned it to the original position.

**Figure 3:**
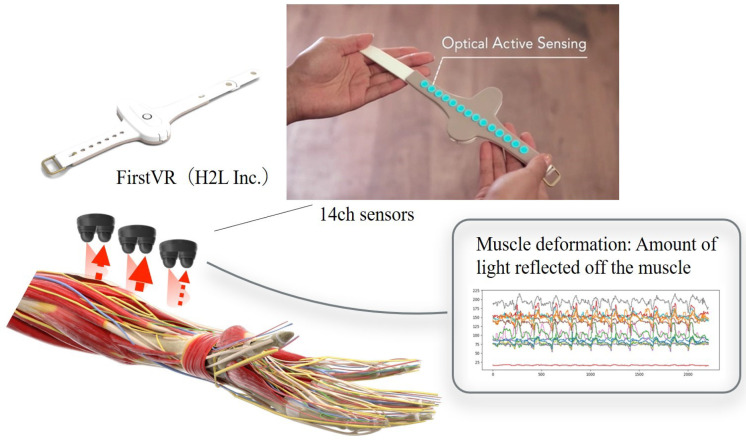
Equipment used in the experiment. The muscle strain sensor is an optical sensor that acquires displacement information of the forearm’s skeletal muscles as time series data. This makes it possible to estimate the force at the fingertips. Additionally, acceleration and quaternions can be acquired.

The range of motion exercises of physiotherapy were the subjects’ tasks during the experiment. Two common joint range-of-motion exercises were established for the task, “upper limb raising exercise” and “lower limb flexion exercise”. During the experiment, subjects used a human model for nursing practice (Figure 2). The experimental tasks were defined by their starting, ending, and reached positions. Each task was executed in two sets of five repetitions (ten trials in total).

(A) Upper limb raising exercise:
  The starting position of the experiment was set with the elbow joint of the human model extended and in contact with the bed. The arrival position was set at the 90° shoulder joint of the human model. The end position of the movement was the same as the start position. The subject was instructed to grasp the following parts of the human model. The subject held the proximal shoulder joint of the human model with the left hand from the ventral side and the distal forearm with the right hand from the dorsal side. The subjects were instructed to position their lower limb for the task so that they were comfortable.
(B) Lower limb flexion exercise:
  The experiment’s starting position was where the lower limb of the human model was in contact with the bed in an extended position. The position reached was 90° hip flexion. The end position was set to the same as the start position. The part of the human body model to be grasped by the subject was specified as follows. The subject grasped the thigh of the human model with the left hand from the dorsal side and the distal shank with the right hand from the ventral side. The position of the subject’s lower limb remained the same as in the upper limb raising exercise. One experimenter experimented on this.
  In this study, an OMG sensor, the muscle deformation sensor array FirstVR (H2L Inc., Tokyo, Japan), shown in Figure 3 [30], was used. The muscle deformation sensor array contains 14 optical muscle deformation sensors. It can optically measure muscle bulge (muscle deformation) and estimate intrinsic sensation. The device is also equipped with a gyro sensor and a 3-axis acceleration sensor, making it possible to acquire quaternion data (posture data of the part of the body wearing the device). The device can be worn by wrapping it around the forearm like a wristwatch to measure muscle deformation in the forearm and fingers [31]. It can also be worn on the lower leg to measure the deformation of the lower leg muscles [32] or placed on the neck to measure the neck muscles [33]. The readings were transmitted from FirstVR to the PC via Bluetooth Low Energy. The sampling frequency for the data recording was approximately 50 Hz.

### 2.3. Analysis Method

#### 2.3.1. Data Pretreatment

**Figure 4:**
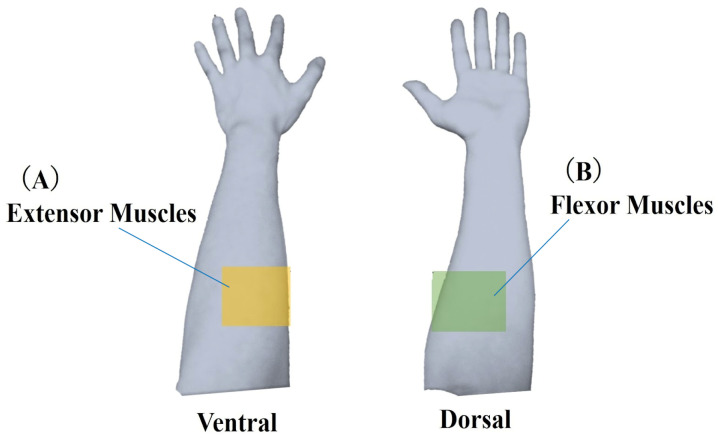
Classification of muscle deformation data. Forearm muscles were categorized into two groups: (**A**) extensor muscles, including ulnar and radial extensors, and (**B**) flexor muscles, including ulnar and radial flexors. Muscle deformation data from five trials were averaged and subjected to further analysis for each task.

In this study, each task’s start and end points were determined based on quaternion data representing the posture of the device-worn region. The raw muscle deformation signals (sampling 50 Hz) were smoothed using a moving average of 100 samples. Moving average processed data were differenced from the starting values and compared as relative data. The 14 optical channels were anatomically divided into extensor muscles (ch0, 1, 3, 12, 13) and flexor muscles (ch4, 6, 7, 9) (Figure 4).

#### 2.3.2. Analysis Method

For each repetition, we first calculated the extensor and flexor autocorrelation coefficients and the cross-correlation coefficient between these muscle groups using Python (ver. 3.10.12). The five coefficients obtained within a set were averaged to yield a set-level value. The two set-level values were then averaged to produce a single representative coefficient per participant. The extreme (non-zero-lag) value of the autocorrelation function was used as an index of periodicity, while cross-correlation values close to +1 or −1 indicated potent agonist–antagonist coupling [34]. Group-level statistical analyses were conducted in R (ver. 4.2.2, New Zealand). The normality of each variable was tested using the Shapiro–Wilk test. Depending on the result, either independent samples *t*-tests or Mann–Whitney U tests were applied for initial comparisons. The Holm–Bonferroni method was used to adjust for multiple comparisons. The significance threshold was set at *p* < 0.05 (adjusted). To account for potential confounding factors such as age, sex, height, and weight, we additionally performed ANCOVA (analysis of covariance) for each outcome. The group effect (novice vs. expert) was assessed while statistically adjusting for these covariates. Partial $\eta^{2}$ values were reported as effect size measures, interpreted using Cohen’s thresholds (small: 0.01; medium: 0.06; large: 0.14). Bootstrapped 95 confidence intervals were also calculated to support the interpretation of non-significant trends. In addition, unadjusted *p*-values were corrected for multiple comparisons using the Benjamini–Hochberg procedure to control for the false discovery rate.

### 2.4. Ethical Considerations

This study was conducted in accordance with the principles of the Declaration of Helsinki. It was also approved by the Ethical Review Committee of The Okinawa Rehabilitation Welfare Institute (approval no. 2021-04). Explanations were provided orally by the experimenter with documented details. If the participant was a student, it was explained that there would be no disadvantage due to academic performance, regardless of whether the participant agreed to participate in the study. The Ethical Review Committee waived the requirement for parental consent for minors’ consent.

## 3. Results

### 3.1. Time Series of Muscle Deformation Data

**Figure 5:**
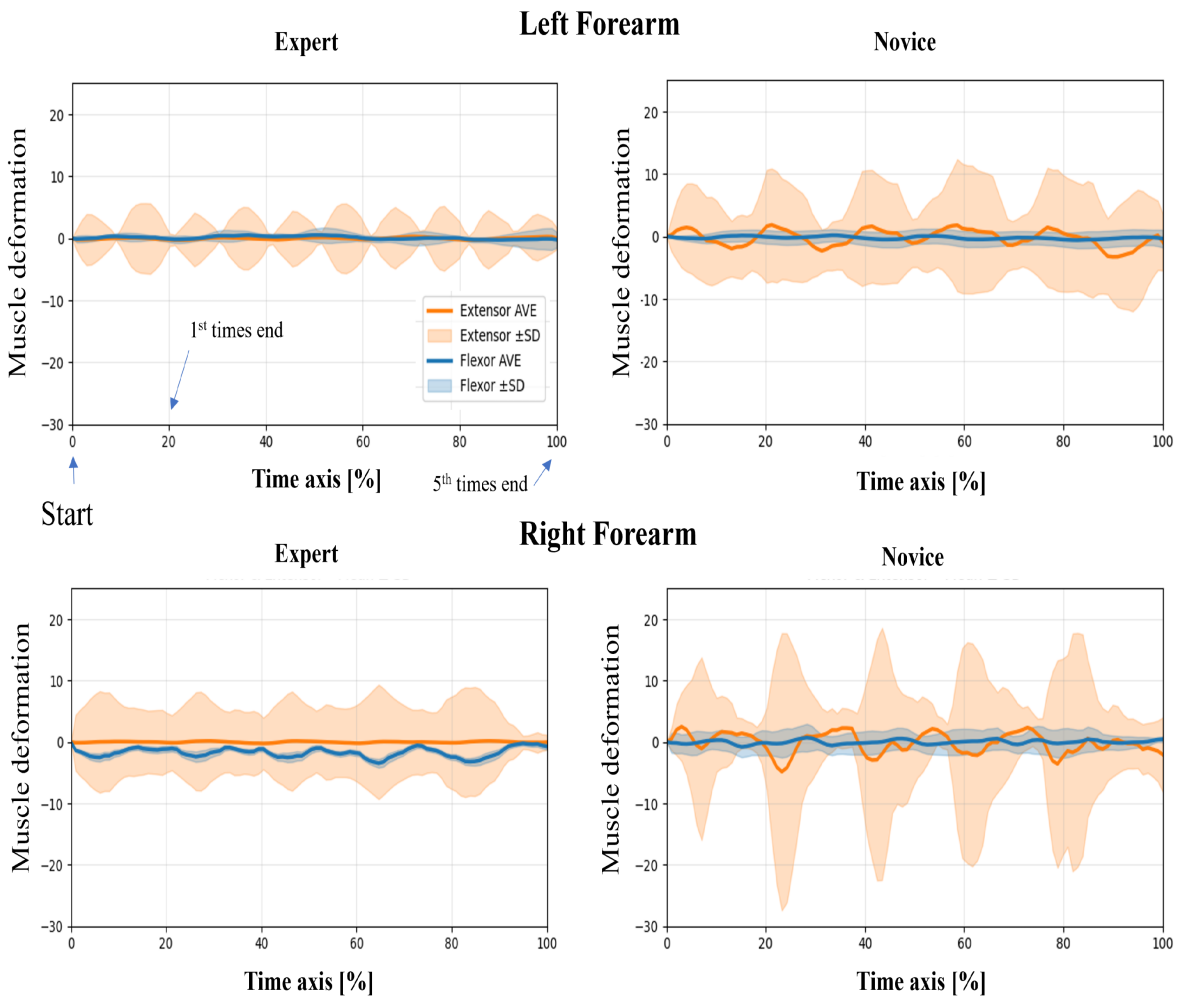
Time-normalized muscle deformation signals of flexor and extensor groups during the upper limb raising task. Each participant performed five repetitions per condition. Time series signals were normalized to 100% and averaged across repetitions. Solid lines represent the group mean (extensor: orange, flexor: blue), and shaded areas indicate ±1 standard deviation (SD) across trials. Plots are separated by group (Expert vs. Novice) and arm (Left vs. Right Forearm).

**Figure 6:**
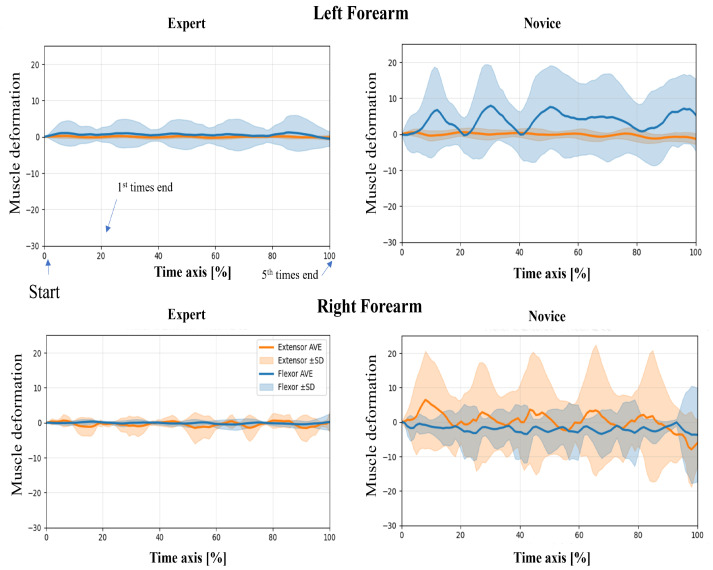
Time-normalized muscle deformation signals of flexor and extensor groups during the lower limb flexion exercise. Each participant performed five repetitions per condition. Time series signals were normalized to 100% and averaged across repetitions. Solid lines represent the group mean (extensor: orange, flexor: blue), and shaded areas indicate ±1 standard deviation (SD) across trials. Plots are separated by group (Expert vs. Novice) and arm (Left vs. Right Forearm). Novice participants show greater temporal variability than experts, particularly in the right forearm.

In this study, wearable muscle deformation sensors were used to record time series data of the forearm muscles of novices and experts. The resulting data were visualized for both tasks, revealing observable differences between the groups in waveform stability, bilateral symmetry, and temporal regularity (Figure 5 and Figure 6). The experts generally exhibited smooth and stable deformation waveforms in both forearm flexor and extensor muscles. In contrast, novices showed greater fluctuations, particularly in the flexor muscles, with irregular periodicity, suggesting less stable muscle control.

Regarding left–proper symmetry, experts showed highly similar deformation patterns between the left and right forearms, indicating balanced bilateral coordination. However, novices demonstrated asymmetric patterns: the flexor muscles in the left forearm exhibited greater deformation, while the extensor muscles in the right forearm showed larger amplitudes. This asymmetry may reflect immature or imbalanced motor control strategies. Regarding temporal patterns, the experts’ muscle activation was rhythmic and repeatable across the five repetitions, suggesting consistent and well-trained motor planning. In contrast, novices exhibited more erratic temporal fluctuations, including abrupt and nonrepetitive changes in amplitude, which may indicate a lack of refined muscle coordination or variability in task execution.

### 3.2. Covariate-Adjusted Group Comparison (ANCOVA)

**Figure 7:**
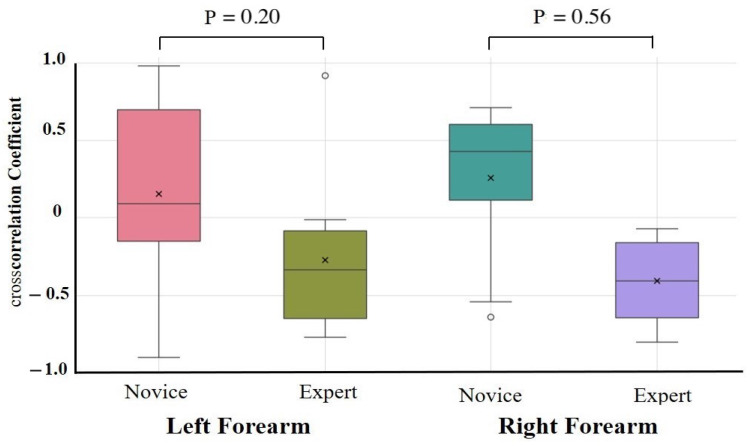
Cross-correlation analysis during upper limb raising exercise. Cross-correlation analysis was performed on forearm flexors and extensors. Although not statistically significant, a moderate-to-large effect size was observed in the forearm during the upper limb raising task, with experts showing more negative cross-correlations than novices.

**Figure 8:**
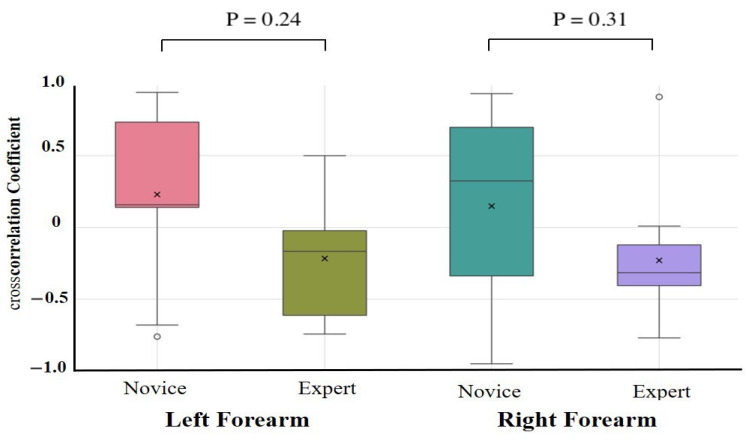
Cross-correlation analysis during lower limb flexion exercise. Cross-correlation analysis was performed on forearm flexors and extensors. Although not statistically significant, a moderate effect size was observed in the forearm during the lower limb flexion task, with experts showing more negative cross-correlations than novices.

The results of the one-way ANCOVAs with Novices and Experts as fixed factors and age, height, weight, and sex as covariates are summarized in Table 2. After adjustment, no autocorrelation coefficient differed significantly between the novices and experts for any task or muscle group (all $p_{\mathrm{adj}}$ > 0.10). The largest residual effect was observed for the right forearm extensor during the lower limb flexion exercise (*F*(1,17) = 2.89, $p_{\mathrm{adj}}$ = 0.11, partial $\eta^{2}$ = 0.18; 95% CI = −0.09 to 0.87), but this was not statistically significant. Unadjusted group comparisons (independent *t*-tests on Fisher-*z*-transformed coefficients) are provided in Supplementary Table S2. These revealed similar effect size trends—in particular, greater right forearm flexor consistency in experts during the upper limb raising task (Novice: 0.32 ± 0.10, Expert: 0.42 ± 0.10, *p* = 0.09, *d* = 0.79)—but likewise did not survive correction for multiple comparisons. Cross-correlation coefficient comparisons were conducted to assess antagonistic muscle coordination (Table 3, Figure 7 and Figure 8). After covariate adjustment, no statistically significant group differences were observed. However, a notable trend was present in the right forearm during the upper limb raising task, with novices showing a positive correlation (0.26 ± 0.50) and experts a negative correlation (−0.41 ± 0.30; $p_{\mathrm{adj}\;}$ = 0.56, partial $\eta^{2}$ = 0.45), suggesting a possible tendency toward greater reciprocal muscle coordination in experts. Moderate effect sizes were also observed for the left and right forearms during the lower limb flexion task ($p_{\mathrm{adj}}$ = 0.24 and 0.31, partial $\eta^{2}$ = 0.19 and 0.12, respectively), although these too were not statistically significant.

Unadjusted group comparisons (independent *t*-tests on Fisher-*z*-transformed coefficients) are provided in Supplementary Table S3. These yielded comparable effect size patterns but did not survive multiple comparison correction.

## 4. Discussion

This study aimed to characterize the differences in muscle activity between experts and novices using time series signals from the forearm muscles acquired via an optical wearable sensor. Visualization, autocorrelation, and cross-correlation analyses were used to assess temporal stability and intermuscular coordination patterns.

Experts generally exhibited smoother and more rhythmic deformation waveforms with reduced variability and clearer periodicity, particularly in the right forearm, than novices. In contrast, novice participants showed greater fluctuations and asymmetry, suggesting a less stable and coordinated motor control. Cross-correlation analysis revealed a significant group difference in the right forearm during upper limb raising, with experts displaying negative correlations between flexors and extensors, indicative of reciprocal activation. In contrast, novices exhibited positive correlations, suggesting inefficient co-contraction.

However, after adjusting for covariates, such as age, height, weight, and sex via ANCOVA, these group differences were no longer statistically significant.These findings suggest that, although experts may adopt more efficient motor strategies, such conclusions should be interpreted cautiously, given the lack of adjusted statistical significance. Given the relatively small sample size (*n* = 10 per group), the study may have lacked sufficient statistical power to detect small-to-moderate effects. This is reflected in the observation of relatively large effect sizes despite non-significant group-level differences. While these trends are encouraging, the limited sample size introduces uncertainty regarding the robustness of the findings and may increase the likelihood of type II errors.

In addition, the absence of multimodal data—particularly neurophysiological signals such as EEG or EMG [35]—limits the ability to cross-validate muscle deformation patterns with other indicators of motor control. As a result, interpretations regarding underlying neural coordination (e.g., cortical or cerebellar mechanisms) remain speculative and should be considered as hypotheses for future investigation rather than definitive conclusions. To enhance the validity and generalizability of future research, it will be important to expand participant diversity and incorporate multimodal measurements that combine wearable sensors with neurophysiological and kinematic data [36].

This study has several methodological limitations. Participant characteristics, such as age, sex, and clinical experience, were not fully matched across groups, although age and body metrics were statistically controlled in the final analyses. The analysis was restricted to two fundamental physiotherapy tasks; thus, the generalizability of the findings to more complex or functional movements remains limited.

As a potential application, we propose developing real-time feedback systems that compare the muscle coordination patterns of novice users with those of expert benchmarks. Such systems can deliver feedback visually or through haptic modalities to support intuitive motor learning, particularly in clinical education. Moreover, recent work has demonstrated that fingertip forces can be estimated using wearable optical and inertial sensors [37], raising the possibility of extending this approach to other domains, such as sports science, human–computer interaction [38], and VR-based training systems [39].

## 5. Conclusions

This study used a wearable muscle deformation sensor array to compare time series muscle activity between novices and experts during two physiotherapy tasks. Autocorrelation and cross-correlation analyses were used to assess muscle control and coordination differences.

Experts generally exhibited more stable, rhythmic, and symmetric muscle deformation patterns, while novices showed greater variability and left–correct asymmetry. Although some group differences in autocorrelation and cross-correlation coefficients reached statistical significance before covariate adjustment, ANCOVA controlling for age, sex, height, and weight revealed no significant group effects. However, several comparisons yielded medium to large effect sizes, indicating potentially significant differences in motor coordination strategies between novices and experts.

These trends support the hypothesis that experts may adopt more efficient and coordinated neuromuscular control patterns. However, further research with larger samples and multimodal validation, such as electromyography and motion tracking, must confirm these preliminary findings. Wearable muscle deformation sensors show promise as objective tools for evaluating motor control in physiotherapy and could be applied to develop real-time feedback systems to support novice training. Beyond rehabilitation, this technology can also contribute to applications in sports science, human–computer interaction, and VR-based skill acquisition environments.

## Figures and Tables

**Table 1:** Attributes of participants.

			Novice (*n* = 10)	Expert (*n* = 10)	*p*-Value
Age	(years old)		19 ± 2.2	39.2 ± 5.2	0.0001
Sex	(male/female)		7/3	9/1	0.5820
Height	(cm)		163.3 ± 9.5	167.6 ± 6.5	0.2564
Body weight	(kg)		57.5 ± 9.2	67.2 ± 12.8	0.0680
Experience	(years)			15.3 ± 3.7	

**Table 2:** Covariate-adjusted ANCOVA results (autocorrelation coefficient).

			Novice (*n* = 10)	Expert (*n* = 10)	*F*	$p_{\mathrm{adj}}$	Partial $\eta^{2}$	95% CI (Lower–Upper)
ULR-ex	Left	EX	0.39 ± 0.2	0.39 ± 0.2	0.05	0.83	0.00	−0.71–0.65
		FL	0.36 ± 0.1	0.39 ± 0.1	0.12	0.74	0.04	−0.30–0.62
	Right	EX	0.40 ± 0.2	0.39 ± 0.1	0.20	0.66	0.04	−0.56–0.34
		FL	0.32 ± 0.1	0.42 ± 0.1	0.06	0.81	0.18	−0.82–0.67
LLF-ex	Left	EX	0.39 ± 0.2	0.41 ± 0.1	0.80	0.39	0.00	−0.27–0.72
		FL	0.36 ± 0.1	0.34 ± 0.2	0.04	0.84	0.06	−0.55–0.56
	Right	EX	0.33 ± 0.2	0.45 ± 0.1	2.89	0.11	0.18	−0.09–0.87
		FL	0.41 ± 0.2	0.46 ± 0.1	0.71	0.42	0.04	−0.22–0.70

ULR-ex: Upper limb raising exercise. LLF-ex: Lower limb flexion exercise. EX: Extension. FL: Flexion. *F*: F-value. *p*: *p*-value. CI: confidence interval. Partial $\eta^{2}$ interpretation: 0.01 = small, 0.06 = medium, 0.14 = large. (Age, height, weight, sex as covariates).

**Table 3:** Covariate-adjusted ANCOVA results (cross-correlation coefficient).

		Novice (*n* = 10)	Expert (*n* = 10)	*F*	$p_{\mathrm{adj}}$	Partial $\eta^{2}$	95% CI (Lower–Upper)
ULR-ex	Left	0.16 ± 0.6	−0.27 ± 0.5	1.84	0.20	0.26	−1.75–0.58
	Right	0.26 ± 0.5	−0.41 ± 0.3	0.35	0.56	0.45	−1.75–1.09
LLF-ex	Left	0.23 ± 0.6	−0.21 ± 0.5	1.52	0.24	0.19	−2.54–0.95
	Right	0.15 ± 0.7	−0.23 ± 0.5	1.11	0.31	0.12	−2.74–0.91

ULR-ex: Upper limb raising exercise. LLF-ex: Lower limb flexion exercise. *F*: F-value. *p*: *p*-value. CI: confidence interval. Partial $\eta^{2}$ interpretation: 0.01 = small, 0.06 = medium, 0.14 = large. (Age, height, weight, sex as covariates).

## Data Availability

The data used to support the findings of this study are available from the corresponding author upon request.

## Supplementary Materials

The following supporting information can be downloaded at https://www.mdpi.com/article/10.3390/s25113507/s1: Table S1: Results of the Shapiro–Wilk test; Table S2: Unadjusted descriptive statistics and pairwise comparisons of autocorrelation coefficients; Table S3: Unadjusted descriptive statistics and pairwise comparisons of cross-correlation coefficients.
