# Supplementary material for: Time Series Analysis of Muscle Deformation During Physiotherapy Using Optical Wearable Sensors
Source: Sensors (Basel). 2025 Jun 2;25(11):3507. doi: 10.3390/s25113507 (PMC12158351; doi:10.3390/s25113507)
Supplement: Supplementary file 1 [file sensors-25-03507-s001.zip › sensors-3584499-supplementary.pdf]

Table S1.Results of the Shapiro–Wilk test

			Novice (n=10)	Shapiro–Wilk p	Expert (n=10)	Shapiro–Wilk p
ULR exercise	Left	EX	0.39 ± 0.2	0.06	0.39 ± 0.2	0.55
		FL	0.36 ± 0.1	0.26	0.39 ± 0.1	0.61
	Right	EX	0.40 ± 0.2	0.97	0.39 ± 0.1	0.99
		FL	0.32 ± 0.1	0.82	0.42 ± 0.1	0.09
LLF exercise	Left	EX	0.39 ± 0.2	0.33	0.41 ± 0.1	0.55
		FL	0.36 ± 0.1	0.67	0.34 ± 0.2	0.61
	Right	EX	0.33 ± 0.2	0.35	0.45 ± 0.1	0.01
		FL	0.41 ± 0.2	0.94	0.46 ± 0.1	0.29

ULR : Upper limb raising , LLF : Lower limb flexion , EX : Extension , FL : Flexion ,
p : p-value ,
In the Shapiro–Wilk test, $p < 0.05$ was considered evidence of non-normality, and independent two-group comparisons were conducted using t-tests or Mann–Whitney U-tests accordingly.

Table S2. Unadjusted descriptive statistics and pairwise comparisons of autocorrelation coefficients

			p	p_adj	Cohen d	Power
ULR exercise	Left	EX	0.97	0.97	0.02	0.05
		FL	0.53	0.85	0.29	0.34
	Right	EX	0.46	0.85	0.34	0.20
		FL	0.09	0.60	0.79	0.58
LLF exercise	Left	EX	0.79	0.91	0.12	0.06
		FL	0.80	0.91	0.11	0.06
	Right	EX	0.15	0.60	0.32	0.18
		FL	0.46	0.85	0.33	0.19

ULR : Upper limb raising , LLF : Lower limb flexion , EX : Extension , FL : Flexion , p : p-value , p_adj： the p-value adjusted for multiple comparisons,

The effect size is specified as 0.1 for small, 0.3 for medium, and 0.5 for large

Table S3. Unadjusted descriptive statistics and pairwise comparisons of cross-correlation coefficients

		p	p_adj	Cohen d	Power
ULR exercise	Left	0.10	0.13	0.33	0.19
	Right	0.01	0.04	0.60	0.57
LLFexercise	Left	0.07	0.13	0.85	0.72
	Right	0.17	0.17	0.63	0.55

ULR : Upper limb raising , LLF : Lower limb flexion , EX : Extension , FL : Flexion ,
p : p-value_original , p_adj： the p-value adjusted for multiple comparisons,

CI : confidence interval ,
The effect size is specified as 0.1 for small, 0.3 for medium, and 0.5 for large
